# Supplementary material for: Modeling glioblastoma heterogeneity as a dynamic network of cell states
Source: Mol Syst Biol. 2021 Sep 16;17(9):e10105. doi: 10.15252/msb.202010105 (PMC8444284; doi:10.15252/msb.202010105)
Supplement: Supplementary file 6 — Source Data for Figure 5 [file MSB-17-e10105-s004.zip › Figure5A_sourcedata/GSEA_3017/nosigdb_stateE.GseaPreranked.1622795031093/RICHARDS_NOWAKOWSKI_SCIENCE_2017_MGE_IPC2_UPREG.html]

Details for gene set RICHARDS\_NOWAKOWSKI\_SCIENCE\_2017\_MGE\_IPC2\_UPREG[GSEA]

|  || Dataset | state63017 |
| Phenotype | NoPhenotypeAvailable |
| Upregulated in class | na\_neg |
| GeneSet | RICHARDS\_NOWAKOWSKI\_SCIENCE\_2017\_MGE\_IPC2\_UPREG |
| Enrichment Score (ES) | -0.6261751 |
| Normalized Enrichment Score (NES) | -3.721651 |
| Nominal p-value | 0.0 |
| FDR q-value | 0.0 |
| FWER p-Value | 0.0 |
Table: GSEA Results Summary

  

Fig 1: Enrichment plot: RICHARDS\_NOWAKOWSKI\_SCIENCE\_2017\_MGE\_IPC2\_UPREG      
 Profile of the Running ES Score & Positions of GeneSet Members on the Rank Ordered List

  

| PROBE | GENE SYMBOL | GENE\_TITLE | RANK IN GENE LIST | RANK METRIC SCORE | RUNNING ES | CORE ENRICHMENT || 1 | CDH2 |  |  | 27 | 0.668 | -0.0378 | No |
| 2 | TMSB4X |  |  | 93 | 0.412 | -0.1360 | No |
| 3 | ST8SIA5 |  |  | 150 | 0.334 | -0.2208 | No |
| 4 | RGS12 |  |  | 153 | 0.330 | -0.2219 | No |
| 5 | DCLK2 |  |  | 169 | 0.318 | -0.2432 | No |
| 6 | CDK6 |  |  | 238 | 0.269 | -0.3469 | No |
| 7 | PFN2 |  |  | 265 | 0.253 | -0.3857 | No |
| 8 | LIN54 |  |  | 271 | -0.250 | -0.3919 | No |
| 9 | TMEM245 |  |  | 272 | -0.250 | -0.3905 | No |
| 10 | ARHGAP11B |  |  | 277 | -0.252 | -0.3952 | No |
| 11 | RTTN |  |  | 295 | -0.257 | -0.4200 | No |
| 12 | YEATS4 |  |  | 300 | -0.258 | -0.4246 | No |
| 13 | RNF168 |  |  | 301 | -0.258 | -0.4231 | No |
| 14 | AHI1 |  |  | 310 | -0.261 | -0.4340 | No |
| 15 | PSIP1 |  |  | 319 | -0.263 | -0.4448 | No |
| 16 | HNRNPDL |  |  | 322 | -0.263 | -0.4463 | No |
| 17 | NCAPH2 |  |  | 331 | -0.264 | -0.4572 | No |
| 18 | HNRNPH1 |  |  | 334 | -0.265 | -0.4587 | No |
| 19 | PAICS |  |  | 336 | -0.265 | -0.4587 | No |
| 20 | MCM5 |  |  | 337 | -0.265 | -0.4571 | No |
| 21 | BRD8 |  |  | 341 | -0.266 | -0.4602 | No |
| 22 | HDAC2 |  |  | 348 | -0.268 | -0.4679 | No |
| 23 | KNTC1 |  |  | 352 | -0.269 | -0.4709 | No |
| 24 | STAG1 |  |  | 360 | -0.271 | -0.4802 | No |
| 25 | POLR2A |  |  | 361 | -0.272 | -0.4786 | No |
| 26 | DLEU2 |  |  | 365 | -0.272 | -0.4816 | No |
| 27 | APOLD1 |  |  | 374 | -0.275 | -0.4924 | No |
| 28 | TMEM237 |  |  | 383 | -0.276 | -0.5031 | No |
| 29 | HNRNPA2B1 |  |  | 391 | -0.279 | -0.5123 | No |
| 30 | CCAR1 |  |  | 396 | -0.282 | -0.5168 | No |
| 31 | PPP2R3C |  |  | 400 | -0.283 | -0.5198 | No |
| 32 | NASP |  |  | 410 | -0.285 | -0.5321 | No |
| 33 | MAGI1 |  |  | 413 | -0.287 | -0.5335 | No |
| 34 | SCLT1 |  |  | 419 | -0.289 | -0.5395 | No |
| 35 | CDK2 |  |  | 422 | -0.290 | -0.5409 | No |
| 36 | SEPT10 |  |  | 431 | -0.293 | -0.5515 | No |
| 37 | MYBL1 |  |  | 432 | -0.294 | -0.5498 | No |
| 38 | ZNF738 |  |  | 437 | -0.295 | -0.5542 | No |
| 39 | HMGXB4 |  |  | 438 | -0.296 | -0.5525 | No |
| 40 | CTCF |  |  | 443 | -0.297 | -0.5569 | No |
| 41 | ZNF43 |  |  | 449 | -0.299 | -0.5629 | No |
| 42 | ARL4C |  |  | 453 | -0.300 | -0.5658 | No |
| 43 | ACYP1 |  |  | 454 | -0.301 | -0.5640 | No |
| 44 | HNRNPD |  |  | 456 | -0.301 | -0.5638 | No |
| 45 | MCM4 |  |  | 457 | -0.301 | -0.5620 | No |
| 46 | UBR7 |  |  | 459 | -0.302 | -0.5618 | No |
| 47 | CNTRL |  |  | 462 | -0.304 | -0.5631 | No |
| 48 | DNMT1 |  |  | 466 | -0.304 | -0.5659 | No |
| 49 | SMC1A |  |  | 472 | -0.307 | -0.5718 | No |
| 50 | UPF3B |  |  | 479 | -0.308 | -0.5793 | No |
| 51 | EXOSC9 |  |  | 481 | -0.308 | -0.5790 | No |
| 52 | RSRC1 |  |  | 494 | -0.312 | -0.5958 | No |
| 53 | NUP205 |  |  | 495 | -0.313 | -0.5939 | No |
| 54 | ILF2 |  |  | 497 | -0.313 | -0.5936 | No |
| 55 | RFC2 |  |  | 498 | -0.313 | -0.5918 | No |
| 56 | CEP97 |  |  | 499 | -0.313 | -0.5899 | No |
| 57 | PIK3R3 |  |  | 508 | -0.317 | -0.6004 | No |
| 58 | BARD1 |  |  | 510 | -0.318 | -0.6001 | No |
| 59 | DNA2 |  |  | 513 | -0.318 | -0.6013 | No |
| 60 | CCDC150 |  |  | 516 | -0.319 | -0.6025 | No |
| 61 | HAUS1 |  |  | 518 | -0.320 | -0.6022 | No |
| 62 | KIF20A |  |  | 523 | -0.321 | -0.6065 | No |
| 63 | NUP62 |  |  | 529 | -0.323 | -0.6123 | No |
| 64 | LSM3 |  |  | 530 | -0.323 | -0.6104 | No |
| 65 | CEP192 |  |  | 531 | -0.324 | -0.6085 | No |
| 66 | CEP135 |  |  | 532 | -0.325 | -0.6066 | No |
| 67 | PRIM1 |  |  | 539 | -0.328 | -0.6139 | No |
| 68 | H3F3A |  |  | 542 | -0.329 | -0.6151 | No |
| 69 | JADE1 |  |  | 543 | -0.329 | -0.6131 | No |
| 70 | DCLRE1C |  |  | 544 | -0.330 | -0.6112 | No |
| 71 | CEP57 |  |  | 545 | -0.330 | -0.6093 | No |
| 72 | MSH2 |  |  | 548 | -0.332 | -0.6104 | No |
| 73 | CDC25C |  |  | 549 | -0.332 | -0.6084 | No |
| 74 | GPSM2 |  |  | 550 | -0.334 | -0.6065 | No |
| 75 | PLK4 |  |  | 551 | -0.334 | -0.6045 | No |
| 76 | HAUS6 |  |  | 555 | -0.337 | -0.6071 | No |
| 77 | ZNF90 |  |  | 557 | -0.338 | -0.6067 | No |
| 78 | SAP30 |  |  | 558 | -0.339 | -0.6047 | No |
| 79 | MCM3 |  |  | 560 | -0.340 | -0.6042 | No |
| 80 | HAT1 |  |  | 561 | -0.340 | -0.6022 | No |
| 81 | TROAP |  |  | 562 | -0.341 | -0.6002 | No |
| 82 | CCDC15 |  |  | 576 | -0.347 | -0.6183 | No |
| 83 | HMGB3 |  |  | 577 | -0.348 | -0.6162 | No |
| 84 | RCC1 |  |  | 582 | -0.350 | -0.6204 | No |
| 85 | GEN1 |  |  | 584 | -0.351 | -0.6198 | No |
| 86 | NUP107 |  |  | 586 | -0.352 | -0.6193 | No |
| 87 | DLL1 |  |  | 588 | -0.352 | -0.6188 | No |
| 88 | DDX39A |  |  | 590 | -0.353 | -0.6182 | No |
| 89 | CDK5RAP2 |  |  | 591 | -0.353 | -0.6162 | No |
| 90 | CENPC |  |  | 596 | -0.356 | -0.6202 | No |
| 91 | RNASEH2B |  |  | 599 | -0.356 | -0.6212 | No |
| 92 | ZNF519 |  |  | 600 | -0.356 | -0.6191 | No |
| 93 | TTF1 |  |  | 602 | -0.358 | -0.6186 | No |
| 94 | HIRIP3 |  |  | 603 | -0.359 | -0.6165 | No |
| 95 | FANCB |  |  | 606 | -0.361 | -0.6174 | No |
| 96 | PRIM2 |  |  | 607 | -0.362 | -0.6153 | No |
| 97 | WDHD1 |  |  | 610 | -0.363 | -0.6162 | No |
| 98 | KIF18B |  |  | 612 | -0.363 | -0.6156 | No |
| 99 | TOPBP1 |  |  | 614 | -0.364 | -0.6150 | No |
| 100 | BTG3 |  |  | 615 | -0.365 | -0.6129 | No |
| 101 | RFWD3 |  |  | 617 | -0.365 | -0.6123 | No |
| 102 | C18orf54 |  |  | 619 | -0.366 | -0.6117 | No |
| 103 | ZGRF1 |  |  | 620 | -0.367 | -0.6095 | No |
| 104 | G2E3 |  |  | 624 | -0.371 | -0.6120 | No |
| 105 | WDR76 |  |  | 626 | -0.373 | -0.6113 | No |
| 106 | LCORL |  |  | 627 | -0.374 | -0.6091 | No |
| 107 | NEK2 |  |  | 628 | -0.374 | -0.6069 | No |
| 108 | EXOSC8 |  |  | 630 | -0.376 | -0.6062 | No |
| 109 | RNASEH2A |  |  | 633 | -0.378 | -0.6071 | No |
| 110 | RMI1 |  |  | 637 | -0.381 | -0.6095 | No |
| 111 | MPHOSPH9 |  |  | 644 | -0.384 | -0.6165 | No |
| 112 | PID1 |  |  | 645 | -0.385 | -0.6142 | No |
| 113 | TTF2 |  |  | 646 | -0.386 | -0.6119 | No |
| 114 | GAS2L3 |  |  | 647 | -0.388 | -0.6096 | No |
| 115 | ZNF704 |  |  | 649 | -0.392 | -0.6089 | No |
| 116 | TMEM106C |  |  | 654 | -0.397 | -0.6127 | No |
| 117 | GINS2 |  |  | 658 | -0.399 | -0.6150 | No |
| 118 | PBX3 |  |  | 662 | -0.403 | -0.6173 | No |
| 119 | HIST1H2BH |  |  | 663 | -0.404 | -0.6149 | No |
| 120 | SAE1 |  |  | 668 | -0.408 | -0.6187 | No |
| 121 | DSN1 |  |  | 671 | -0.410 | -0.6193 | No |
| 122 | KIF18A |  |  | 673 | -0.412 | -0.6185 | No |
| 123 | NUDT1 |  |  | 674 | -0.413 | -0.6160 | No |
| 124 | CIT |  |  | 680 | -0.418 | -0.6213 | No |
| 125 | H2AFV |  |  | 684 | -0.423 | -0.6234 | No |
| 126 | CENPO |  |  | 686 | -0.424 | -0.6225 | No |
| 127 | CENPQ |  |  | 689 | -0.425 | -0.6231 | No |
| 128 | STIL |  |  | 692 | -0.425 | -0.6237 | Yes |
| 129 | CKAP5 |  |  | 694 | -0.426 | -0.6227 | Yes |
| 130 | DIAPH3 |  |  | 696 | -0.427 | -0.6217 | Yes |
| 131 | ANP32B |  |  | 699 | -0.431 | -0.6223 | Yes |
| 132 | E2F3 |  |  | 700 | -0.431 | -0.6197 | Yes |
| 133 | CENPL |  |  | 703 | -0.432 | -0.6203 | Yes |
| 134 | CHEK1 |  |  | 704 | -0.433 | -0.6177 | Yes |
| 135 | PARPBP |  |  | 705 | -0.434 | -0.6152 | Yes |
| 136 | TIMELESS |  |  | 707 | -0.435 | -0.6141 | Yes |
| 137 | POLD3 |  |  | 710 | -0.437 | -0.6147 | Yes |
| 138 | RBBP8 |  |  | 714 | -0.440 | -0.6167 | Yes |
| 139 | RAD18 |  |  | 715 | -0.443 | -0.6141 | Yes |
| 140 | ALYREF |  |  | 716 | -0.444 | -0.6115 | Yes |
| 141 | SKA1 |  |  | 719 | -0.447 | -0.6119 | Yes |
| 142 | MASTL |  |  | 721 | -0.451 | -0.6108 | Yes |
| 143 | TTK |  |  | 724 | -0.454 | -0.6112 | Yes |
| 144 | POLQ |  |  | 726 | -0.455 | -0.6101 | Yes |
| 145 | LINC01224 |  |  | 727 | -0.455 | -0.6074 | Yes |
| 146 | RAD21 |  |  | 728 | -0.458 | -0.6047 | Yes |
| 147 | SPAG5 |  |  | 729 | -0.458 | -0.6020 | Yes |
| 148 | CCNF |  |  | 732 | -0.460 | -0.6024 | Yes |
| 149 | POLA1 |  |  | 733 | -0.460 | -0.5997 | Yes |
| 150 | BRIP1 |  |  | 734 | -0.461 | -0.5969 | Yes |
| 151 | GAD1 |  |  | 737 | -0.461 | -0.5973 | Yes |
| 152 | LBR |  |  | 739 | -0.463 | -0.5961 | Yes |
| 153 | HIST1H3D |  |  | 740 | -0.467 | -0.5934 | Yes |
| 154 | E2F8 |  |  | 741 | -0.467 | -0.5906 | Yes |
| 155 | MGME1 |  |  | 742 | -0.468 | -0.5878 | Yes |
| 156 | SMC3 |  |  | 743 | -0.470 | -0.5851 | Yes |
| 157 | NUCKS1 |  |  | 744 | -0.471 | -0.5823 | Yes |
| 158 | PTMA |  |  | 745 | -0.475 | -0.5795 | Yes |
| 159 | CHD7 |  |  | 746 | -0.480 | -0.5767 | Yes |
| 160 | TK1 |  |  | 747 | -0.480 | -0.5738 | Yes |
| 161 | MIS18A |  |  | 750 | -0.483 | -0.5741 | Yes |
| 162 | LIG1 |  |  | 752 | -0.485 | -0.5727 | Yes |
| 163 | PRR11 |  |  | 755 | -0.488 | -0.5730 | Yes |
| 164 | CDCA3 |  |  | 759 | -0.497 | -0.5747 | Yes |
| 165 | MMS22L |  |  | 761 | -0.500 | -0.5733 | Yes |
| 166 | GMNN |  |  | 766 | -0.510 | -0.5764 | Yes |
| 167 | RTKN2 |  |  | 767 | -0.511 | -0.5734 | Yes |
| 168 | DTYMK |  |  | 774 | -0.522 | -0.5796 | Yes |
| 169 | RFC4 |  |  | 777 | -0.527 | -0.5796 | Yes |
| 170 | STMN1 |  |  | 778 | -0.529 | -0.5765 | Yes |
| 171 | DEPDC1B |  |  | 779 | -0.533 | -0.5733 | Yes |
| 172 | GLCCI1 |  |  | 780 | -0.535 | -0.5702 | Yes |
| 173 | SKA3 |  |  | 783 | -0.539 | -0.5701 | Yes |
| 174 | NCAPD2 |  |  | 784 | -0.540 | -0.5669 | Yes |
| 175 | CEP152 |  |  | 785 | -0.542 | -0.5637 | Yes |
| 176 | BLM |  |  | 786 | -0.543 | -0.5605 | Yes |
| 177 | FANCD2 |  |  | 787 | -0.544 | -0.5573 | Yes |
| 178 | NCAPD3 |  |  | 790 | -0.559 | -0.5571 | Yes |
| 179 | ATAD5 |  |  | 791 | -0.559 | -0.5538 | Yes |
| 180 | HAUS8 |  |  | 792 | -0.560 | -0.5505 | Yes |
| 181 | CDC20 |  |  | 793 | -0.562 | -0.5472 | Yes |
| 182 | DSEL |  |  | 794 | -0.563 | -0.5438 | Yes |
| 183 | HMGN2 |  |  | 795 | -0.564 | -0.5405 | Yes |
| 184 | RACGAP1 |  |  | 796 | -0.566 | -0.5372 | Yes |
| 185 | HMGB1 |  |  | 797 | -0.567 | -0.5338 | Yes |
| 186 | CENPJ |  |  | 799 | -0.568 | -0.5320 | Yes |
| 187 | RFC3 |  |  | 801 | -0.569 | -0.5302 | Yes |
| 188 | TUBB |  |  | 802 | -0.570 | -0.5268 | Yes |
| 189 | TUBB4B |  |  | 804 | -0.573 | -0.5250 | Yes |
| 190 | CENPN |  |  | 805 | -0.574 | -0.5216 | Yes |
| 191 | USP1 |  |  | 806 | -0.575 | -0.5182 | Yes |
| 192 | CDCA8 |  |  | 808 | -0.585 | -0.5163 | Yes |
| 193 | CHAF1A |  |  | 810 | -0.588 | -0.5144 | Yes |
| 194 | DEK |  |  | 812 | -0.591 | -0.5124 | Yes |
| 195 | FOXM1 |  |  | 814 | -0.593 | -0.5105 | Yes |
| 196 | DEPDC1 |  |  | 815 | -0.596 | -0.5070 | Yes |
| 197 | CDCA2 |  |  | 816 | -0.597 | -0.5034 | Yes |
| 198 | MCM7 |  |  | 817 | -0.599 | -0.4999 | Yes |
| 199 | LMNB1 |  |  | 818 | -0.606 | -0.4963 | Yes |
| 200 | MCM8 |  |  | 819 | -0.607 | -0.4927 | Yes |
| 201 | MND1 |  |  | 821 | -0.615 | -0.4907 | Yes |
| 202 | CKAP2L |  |  | 826 | -0.625 | -0.4932 | Yes |
| 203 | AURKB |  |  | 829 | -0.634 | -0.4925 | Yes |
| 204 | HELLS |  |  | 830 | -0.635 | -0.4888 | Yes |
| 205 | DTL |  |  | 831 | -0.637 | -0.4850 | Yes |
| 206 | PCNA |  |  | 832 | -0.640 | -0.4812 | Yes |
| 207 | BUB3 |  |  | 834 | -0.641 | -0.4790 | Yes |
| 208 | CENPW |  |  | 835 | -0.643 | -0.4752 | Yes |
| 209 | CDKN3 |  |  | 836 | -0.653 | -0.4713 | Yes |
| 210 | BUB1 |  |  | 837 | -0.653 | -0.4675 | Yes |
| 211 | ITGB3BP |  |  | 838 | -0.661 | -0.4636 | Yes |
| 212 | E2F7 |  |  | 840 | -0.675 | -0.4611 | Yes |
| 213 | ANP32E |  |  | 841 | -0.676 | -0.4571 | Yes |
| 214 | KIF22 |  |  | 842 | -0.678 | -0.4531 | Yes |
| 215 | BIRC5 |  |  | 843 | -0.679 | -0.4491 | Yes |
| 216 | CENPM |  |  | 844 | -0.682 | -0.4451 | Yes |
| 217 | NCAPH |  |  | 845 | -0.685 | -0.4411 | Yes |
| 218 | BRCA1 |  |  | 846 | -0.691 | -0.4370 | Yes |
| 219 | ZWINT |  |  | 847 | -0.692 | -0.4329 | Yes |
| 220 | SPC24 |  |  | 848 | -0.694 | -0.4288 | Yes |
| 221 | BRCA2 |  |  | 850 | -0.694 | -0.4263 | Yes |
| 222 | DHFR |  |  | 851 | -0.701 | -0.4221 | Yes |
| 223 | VRK1 |  |  | 852 | -0.711 | -0.4179 | Yes |
| 224 | ECT2 |  |  | 854 | -0.717 | -0.4152 | Yes |
| 225 | KIF2C |  |  | 855 | -0.721 | -0.4110 | Yes |
| 226 | NUF2 |  |  | 856 | -0.721 | -0.4067 | Yes |
| 227 | CENPK |  |  | 857 | -0.722 | -0.4025 | Yes |
| 228 | FAM111A |  |  | 858 | -0.727 | -0.3982 | Yes |
| 229 | SYNE2 |  |  | 859 | -0.728 | -0.3939 | Yes |
| 230 | ANLN |  |  | 860 | -0.733 | -0.3895 | Yes |
| 231 | KIF4A |  |  | 861 | -0.736 | -0.3852 | Yes |
| 232 | FEN1 |  |  | 862 | -0.736 | -0.3808 | Yes |
| 233 | FBXO5 |  |  | 864 | -0.742 | -0.3780 | Yes |
| 234 | CCNB2 |  |  | 865 | -0.745 | -0.3736 | Yes |
| 235 | RRM1 |  |  | 866 | -0.749 | -0.3692 | Yes |
| 236 | ARHGAP11A |  |  | 867 | -0.756 | -0.3647 | Yes |
| 237 | NDC80 |  |  | 869 | -0.762 | -0.3618 | Yes |
| 238 | ORC6 |  |  | 870 | -0.766 | -0.3573 | Yes |
| 239 | BUB1B |  |  | 871 | -0.766 | -0.3527 | Yes |
| 240 | CCNA2 |  |  | 872 | -0.770 | -0.3482 | Yes |
| 241 | KIF23 |  |  | 873 | -0.772 | -0.3436 | Yes |
| 242 | MELK |  |  | 874 | -0.776 | -0.3390 | Yes |
| 243 | TMPO |  |  | 875 | -0.778 | -0.3344 | Yes |
| 244 | KIF20B |  |  | 876 | -0.779 | -0.3298 | Yes |
| 245 | CKAP2 |  |  | 878 | -0.781 | -0.3268 | Yes |
| 246 | NCAPG2 |  |  | 879 | -0.781 | -0.3222 | Yes |
| 247 | PTTG1 |  |  | 880 | -0.791 | -0.3175 | Yes |
| 248 | MCM10 |  |  | 881 | -0.798 | -0.3128 | Yes |
| 249 | KIFC1 |  |  | 883 | -0.807 | -0.3096 | Yes |
| 250 | KIF14 |  |  | 885 | -0.809 | -0.3063 | Yes |
| 251 | EZH2 |  |  | 886 | -0.810 | -0.3016 | Yes |
| 252 | FAM111B |  |  | 888 | -0.822 | -0.2983 | Yes |
| 253 | KIF15 |  |  | 890 | -0.834 | -0.2949 | Yes |
| 254 | CDCA5 |  |  | 892 | -0.841 | -0.2915 | Yes |
| 255 | H2AFZ |  |  | 893 | -0.849 | -0.2864 | Yes |
| 256 | RAD51AP1 |  |  | 894 | -0.853 | -0.2814 | Yes |
| 257 | KPNA2 |  |  | 895 | -0.858 | -0.2763 | Yes |
| 258 | DBF4 |  |  | 896 | -0.864 | -0.2712 | Yes |
| 259 | SMC2 |  |  | 897 | -0.867 | -0.2661 | Yes |
| 260 | CENPE |  |  | 899 | -0.873 | -0.2625 | Yes |
| 261 | PBK |  |  | 900 | -0.880 | -0.2573 | Yes |
| 262 | FANCI |  |  | 901 | -0.882 | -0.2521 | Yes |
| 263 | SMC4 |  |  | 902 | -0.886 | -0.2469 | Yes |
| 264 | DUT |  |  | 903 | -0.887 | -0.2416 | Yes |
| 265 | MIS18BP1 |  |  | 904 | -0.889 | -0.2364 | Yes |
| 266 | C21orf58 |  |  | 905 | -0.893 | -0.2311 | Yes |
| 267 | CCNB1 |  |  | 906 | -0.895 | -0.2258 | Yes |
| 268 | CCNE2 |  |  | 907 | -0.895 | -0.2205 | Yes |
| 269 | XRCC2 |  |  | 908 | -0.907 | -0.2152 | Yes |
| 270 | CKS1B |  |  | 909 | -0.912 | -0.2098 | Yes |
| 271 | HJURP |  |  | 910 | -0.921 | -0.2044 | Yes |
| 272 | CKS2 |  |  | 911 | -0.922 | -0.1989 | Yes |
| 273 | GTSE1 |  |  | 912 | -0.941 | -0.1934 | Yes |
| 274 | KIF11 |  |  | 913 | -0.957 | -0.1877 | Yes |
| 275 | MYBL2 |  |  | 914 | -0.959 | -0.1821 | Yes |
| 276 | CLSPN |  |  | 916 | -0.964 | -0.1779 | Yes |
| 277 | TYMS |  |  | 917 | -0.965 | -0.1722 | Yes |
| 278 | CENPU |  |  | 918 | -0.971 | -0.1665 | Yes |
| 279 | PRC1 |  |  | 919 | -0.980 | -0.1607 | Yes |
| 280 | MAD2L1 |  |  | 921 | -0.990 | -0.1564 | Yes |
| 281 | UBE2T |  |  | 922 | -0.994 | -0.1505 | Yes |
| 282 | DLGAP5 |  |  | 923 | -0.998 | -0.1446 | Yes |
| 283 | SPC25 |  |  | 924 | -1.033 | -0.1385 | Yes |
| 284 | HIST1H1E |  |  | 925 | -1.049 | -0.1323 | Yes |
| 285 | ESCO2 |  |  | 926 | -1.067 | -0.1260 | Yes |
| 286 | UBE2C |  |  | 928 | -1.108 | -0.1210 | Yes |
| 287 | NUSAP1 |  |  | 929 | -1.112 | -0.1145 | Yes |
| 288 | TPX2 |  |  | 930 | -1.124 | -0.1078 | Yes |
| 289 | NCAPG |  |  | 931 | -1.142 | -0.1011 | Yes |
| 290 | ASPM |  |  | 932 | -1.146 | -0.0943 | Yes |
| 291 | ATAD2 |  |  | 933 | -1.162 | -0.0875 | Yes |
| 292 | CENPF |  |  | 935 | -1.200 | -0.0819 | Yes |
| 293 | HIST1H1C |  |  | 936 | -1.220 | -0.0747 | Yes |
| 294 | HMGB2 |  |  | 937 | -1.238 | -0.0674 | Yes |
| 295 | CDK1 |  |  | 938 | -1.248 | -0.0601 | Yes |
| 296 | RRM2 |  |  | 940 | -1.403 | -0.0533 | Yes |
| 297 | MKI67 |  |  | 941 | -1.407 | -0.0450 | Yes |
| 298 | TOP2A |  |  | 942 | -1.417 | -0.0366 | Yes |
| 299 | HIST1H1D |  |  | 944 | -1.810 | -0.0275 | Yes |
| 300 | HIST1H1B |  |  | 945 | -1.903 | -0.0163 | Yes |
| 301 | HIST1H4C |  |  | 946 | -2.752 | -0.0000 | Yes |
Table: GSEA details [plain text format]

  

Fig 2: RICHARDS\_NOWAKOWSKI\_SCIENCE\_2017\_MGE\_IPC2\_UPREG: Random ES distribution      
 Gene set null distribution of ES for **RICHARDS\_NOWAKOWSKI\_SCIENCE\_2017\_MGE\_IPC2\_UPREG**
